# Boosting curcumin activity against human prostatic cancer PC3 cells by utilizing scorpion venom conjugated phytosomes as promising functionalized nanovesicles

**DOI:** 10.1080/10717544.2022.2048133

**Published:** 2022-03-10

**Authors:** Mohammed W. Al-Rabia, Nabil A. Alhakamy, Waleed Y. Rizg, Adel F. Alghaith, Osama A. A. Ahmed, Usama A. Fahmy

**Affiliations:** a Department of Medical Microbiology and Parasitology, Faculty of Medicine, King Abdulaziz University, Jeddah, Saudi Arabia; b Department of Pharmaceutics, Faculty of Pharmacy, King Abdulaziz University, Jeddah, Saudi Arabia; c Center of Excellence for Drug Research and Pharmaceutical Industries, Faculty of Pharmacy, King Abdulaziz University, Jeddah, Saudi Arabia; d Mohamed Saeed Tamer Chair for Pharmaceutical Industries, Faculty of Pharmacy, King Abdulaziz University, Jeddah, Saudi Arabia; e Department of Pharmaceutics, College of Pharmacy, King Saud University, Riyadh, Saudi Arabia

**Keywords:** Phytosome, prostate cancer, cell cycle, apoptosis, membrane potential

## Abstract

Prostate cancer (PC) is emerging as one of the leading causes of mortality and morbidity worldwide. Curcumin (CUR) is a well-known phytochemical, and scorpion venom (SV) is a natural peptide with proven anticancer properties. However, these natural bioactive agents are limited by low solubility, low bioavailability, poor thermal stability, and short half-lives. Therefore, the aim of this study was to fabricate SV-conjugated CUR phytosomes as promising functionalized nanovesicles and assess their anticancer efficacy in human prostatic cancer PC3 cells. CUR-Phytosome-SV was fabricated using experimental design software in which the zeta potential and particle sizes were used as dependent variables. The anticancer effect of the fabricated formulation was determined by performing a tetrazolium (MTT) assay, cell cycle analysis, annexin V staining, and examining the expression levels of Bcl-associated X-protein (Bax), p53, caspase-3, B-cell lymphoma 2 (Bcl-2), nuclear factor kappa beta (NF-kB), and tumor necrosis factor alpha (TNF-α). The particle size of the nanoconjugates was found to be in the range of 137.5 ± 7.9 to 298.4 ± 11.9 nm, and the zeta potential was 2.9 ± 0.1 to 26.9 ± 1.2 mV. The outcome of the MTT assay showed that curcumin–Phospholipon^®^–scorpion venom (CUR–PL–SV) exhibited a satisfactory level of cytotoxicity, and the IC_50_ was found to be lower than CUR and PL-SV individually. Cell cycle analysis showed predominantly cell cycle arrest at the G2-M and pre-G1 phases. In contrast, annexin V staining showed significant early and late apoptosis events in addition to increased necrosis when PC3 cells were treated with CUR–PL–SV. Reverse-transcriptase polymerase chain reaction (RT-PCR) analysis showed a reduction in expression of Bax, p53, caspase-3, NF-kB, TNF-α, and an increase in Bcl-2 expression. Moreover, a MMP analysis showed a reduction in mitochondrial permeability and hence confirmed the superior anticancer potential of CUR–PL–SV. Thus, the present study showed significant anticancer potency of SV-conjugated CUR phytosomes against human prostatic cancer PC3 cells, making it a novel treatment approach for PC.

## 1. Introduction

Prostate cancer (PC) is the third most frequently diagnosed cancer after lung and colorectal cancers. PC accounts for 7.3% of total diagnosed cancer types, whereas lung and colorectal account for 11.4% and 10.0%, respectively (Sung et al., 2021). PC is more prevalent in countries with a higher human development index (HDI) than lower index (37.5 and 11.3/100,000 individuals, respectively). Geographically, Western and Northern Europe, North America, New Zealand, and Australia account for a higher number of cases, whereas Asian countries, such as Japan and Singapore, account for the lower cases (Sung et al., 2021). Altered function of androgen receptors is considered the hallmark of PC (Evangelista et al., 2021). Clinical and preclinical studies have also shown the involvement of multiple pathogenic factors, such as androgen mutations, amplification, altered androgen post-translational modifications, intertumoral synthesis of androgens, and dysregulation of c-MYC, phosphoinositide 3 kinase/protein kinase B/phosphatase and tensin homolog (PI3k/Akt/PTEN), p53/p38, and mitogen-activated protein kinase (MAPK) pathways (Wang et al., 2018). Based on disease severity, radiotherapy, surgery, chemotherapy, and immunotherapy can be used as treatment options. Abiraterone acetate, docetaxel, goserelin, leuprolide, buserelin, enzalutamide, darolutamide, flutamide, apalutamide, flutamide, bicalutamide, olaparib, and sipuleucel-T are some of the commonly used pharmacotherapeutic agents (Litwin & Tan, 2017). However, despite being potent drug candidates, the use of these drugs is associated with variable degrees of toxicity and unsatisfactory endpoints.

Curcumin (CUR) is one of the extensively studied phytochemicals and is extracted from *Curcuma longa* rhizomes. CUR is characterized as ‘Generally Recognized as Safe (GRAS)’ by the United States Food and Drug Administration (US FDA; Perera et al., 2020). Pharmacological studies conducted to date report antioxidant, anti-inflammatory, neuroprotective, cardioprotective, reno-protective, antiviral, antifungal, antiproliferative, and anticancer properties of CUR (Liczbiński et al., 2020). In various *in vitro* and *in vivo* studies, CUR showed inhibition of tumorigenesis, initiation, proliferation, metastasis, and angiogenesis (Baldi et al., 2020). Mechanistically, CUR inhibits the progression of the cell cycle, induces apoptosis, alters mitochondrial membrane permeability, inhibits angiogenesis mechanism, and inhibits the proliferative pathways. Moreover, CUR has also been reported to reverse altered signaling pathways, such as PI3K/Akt, p53/p38 MAPK, and others toward normal, and thus, more than 60 clinical trials for the anticancer role of CUR have been registered (Baldi et al., 2020).

Considering the role of CUR in PC, several studies have reported inhibition of proliferation, cell cycle arrest, anti-angiogenic properties, and induction of apoptosis (Hong et al., 2006; Teiten et al., 2010; Yallapu et al., 2012). Despite being a potent phytochemical, CUR suffers from the limitation of low solubility, low bioavailability, and rapid hepatic metabolism (Teiten et al., 2010). In one of the clinical studies, when 10 and 20 g of CUR were administered orally, the maximum concentration was found to be 2.30 ± 0.26 and 1.73 ± 0.19 μg.mL^−1^, respectively. In other clinical studies, 3.6 g of CUR daily for four months showed no effect on tumorigenesis markers (Hong et al., 2006). Thus, based on these studies and various others, it was concluded that CUR undergoes extensive metabolism and hence, needs some technological advancement for optimizing clinical benefits.

Recently, nanocarrier-based drug delivery systems have attracted significant attention for use in cancer therapy (Jain et al., 2010). Among various nanocarriers, phyto-phospholipid complexes (phytosomes) have attracted widespread attention in overcoming the pharmacokinetic limitations of phytoconstituents (Jain et al., 2010). Phytosomes are prepared by using a complex mixture of phytoconstituents and phospholipids under well-defined conditions (Pastorelli et al., 2018). Generally, amphipathic phospholipids, such as phosphatidylethanolamine, phosphatidic acid, phosphatidylglycerol, and phosphatidylcholine, are used to incorporate the drugs so that the resulting complex can easily cross the gastrointestinal (GI) membrane (Li et al., 2015; Gnananath et al., 2017). These phytosomes are easily absorbed, exhibit significantly higher bioavailability, and ultimately produce improved pharmacological outcomes. Moreover, phytosomes significantly differ from liposomes because drugs are dispersed inside the cavity or in the membranous layer in liposomes. In contrast, in the case of phytosomes, drugs are an integral part of the membrane (Pastorelli et al., 2018).

Peptide-conjugated dosage forms have attracted attention in cancer therapy (Hawryłkiewicz & Ptaszyńska, 2021; Li et al., 2021). Scorpion venom (SV) is a diverse and pleiotropic natural peptide and possesses potent anticancer properties (Alhakamy et al., 2020a). SV consists of various peptides that are a rich source of disulfide bonds, are stable, and exhibit a multifactorial mechanism of action (Gómez Rave et al., 2019). In PC, dysregulated ion channels have been reported, and SV is well-reported to reverse this dysregulation toward normal (Akef et al., 2017). Additionally, SV is a potent inductor of apoptosis and inhibitor of proliferation, angiogenesis, and metastasis. However, unlike most natural bioactive agents, SV also suffers from low stability limitations, undergoes thermal degradation, is metabolized, and possess a very short half-life (Akef et al., 2017).

Therefore, in the present study, SV-conjugated CUR phytosomes were fabricated and optimized. The resulting phytosome complex was characterized in terms of particle size and zeta potential, and the success of the formulation was validated by exploring the anticancer potential in human prostatic cancer PC3 cells (Kaighn et al., 1979; Tai et al., 2011).

## 2. Materials and methods

### 2.1. Materials and reagents

In this study, SV (chlorotoxin from *Leiurus quinquestriatus* (North Africa) ≥98% HPLC) and CUR (≥94% (curcuminoid content), ≥80% CUR), were purchased from Sigma-Aldrich Incorporation (St. Louis, MO) and used accordingly. As a gift, Germany-based Lipoid GmbH (Ludwigshafen, Germany) provided Phospholipon^®^ 90H (PL). PL is a hydrogenated phosphatidylcholine with 90% content of soybean origin. Egypt-based VACSERA provided the human tumor cells (PC3), used in this study, via a human prostatic adenocarcinoma metastatic to bone although the US-based ATCC was the organization from which it was originally acquired. The study also purchased fetal bovine serum (FBS), Dulbecco’s modified Eagle medium (DMEM), in addition to other materials for culture from Thermo Fisher Scientific (Waltham, MA).

### 2.2. Curcumin–Phospholipon^®^–scorpion venom (CUR–PL–SV) nano-phytosome design and optimization

To formulate and optimize CUR–PL–SV nanovesicles, a factorial experimental design was utilized in this study. Concentrations of PL (µM, *X*_1_) and concentrations of SV (µM, *X*_2_) were the independent variables. Vesicle sizes were the response parameters. The coded levels (−1, 0, +1) and their values are indicated in Table 1. Nine formulations were generated by the design as indicated in Table 2. Design-Expert 12 was the software that statistically analyzed the responses data. To select the most suitable sequential model for all responses, model fit statistics was utilized on the basis of *R*^2^, the forecasted/adjusted coefficients, and adequate precision ratios and predicted residuals sum of squares (PRESS). This step was followed by the process of generating the optimal model equations for the investigated responses, and the use of analysis of variance (ANOVA) to estimate the significance (at *p*<.05) of the responses’ variables along with their potential interactions.

**Table 1.** Independent variables’ levels and responses’ constraints utilized in the 3^2^ factorial design for the optimization of Curcumin–Phospholipon^®^–Scorpion venom (CUR–PL–SV) phytosomes.

Independent variables	Levels		
	(−1)	(0)	(+1)
*X*_1_: PL concentration (µM)	1.0	2.0	3.0
*X*_2_: SV concentration (µM)	0.1	0.3	0.5

Dependent variables	Desirability constraint
*Y*_1_: vesicle size (nm)	Minimize
*Y*_2_: zeta potential (mV)	Maximize

CUR: curcumin; PL: Phospholipon^®^ 90H; SV: scorpion venom peptide.

**Table 2.** Combination of independent variables in CUR–PL–SV phytosomes experimental runs prepared according to 3^2^ factorial design and their corresponding responses.

Run	*X*_1_	*X*_2_	*Y*_1_	*Y*_2_
	PL concentration (µM)	SV concentration (µM)	Vesicle size^a^±SD (nm)	Zeta potential^a^±SD (mV)
1	2	0.1	211.3 ± 8.9	4.6 ± 0.2
2	3	0.1	276.8 ± 11.3	2.9 ± 0.1
3	1	0.3	170.5 ± 5.8	19.8 ± 0.9
4	2	0.3	233.4 ± 8.4	11.6 ± 0.3
5	3	0.5	318.2 ± 12.3	21.6 ± 0.8
6	2	0.5	255.2 ± 10.2	18.9 ± 0.7
7	1	0.5	199.6 ± 9.2	26.9 ± 1.2
8	1	0.1	137.5 ± 7.9	8.9 ± 0.2
9	3	0.3	298.4 ± 11.9	9.8 ± 0.3

CUR: curcumin; PL: Phospholipon^®^ 90H; SV: scorpion venom peptide; SD: standard deviation.

a Results are presented as mean ± SD, *n* = 5.

### 2.3. Preparation of CUR–PL–SV nanovesicles

CUR–PL–SV were prepared as previously described with several modifications (Alhakamy et al., 2020b). Briefly, CUR (30 mg) and PL (design-based amount) were added to 25 mL dichloromethane. After refluxing this organic solution at 60 °C, the solution was subject to evaporation from 5 mL concentrate and then to lyophilization for a period of 72 h with the view to deriving CUR-PL dried mass. Separately, distilled water (design-based amount) was used to dissolve SV. The dried CUR-PL mass was hydrated using the aqueous SV solution for preparing CUR–PL–SV nanovesicles (Alhakamy et al., 2020b).

### 2.4. Potential measurement of CUR–PL–SV

This study determined the zeta potential and size of CUR–PL–SV nanovesicles after using double distilled water to dilute these vesicles. The average of five replicates was calculated to express the results.

### 2.5. Optimization of CUR–PL–SV nanovesicles

As Table 1 shows, the desirability function was calculated to predict the factors optimized levels to maximize the zeta potential and obtain the minimum size. This step was followed by the preparation of CUR–PL–SV nanovesicles (optimized), and a comparison was drawn between the predicted zeta data/size with the actual results.

### 2.6. Evaluation of anticancer potential of CUR–PL–SV

#### 2.6.1. Optimized CUR–PL–SV PC3 cytotoxicity

The tetrazolium (MTT) assay was used for a cytotoxicity study of optimized CUR–PL–SV in the PC3 cells, which were maintained in DMEM that consisted of penicillin-streptomycin, 10% FBS, sodium pyruvate, and l-glutamine. Cells were grown (5 × 10^3^ cells per well) in 96-well plates and incubated overnight. These PC3 cells were treated using PL-SV, CUR-raw, or CUR–PL–SV. After 48 h incubation, a MTT solution (10 µL) was used to treat these cells following the incubation for 4 h at 37 °C. Formosan crystals were dissolved and assessed at 570 nm using a microplate reader. Non-linear regression analysis (GraphPad Prism software v8, La Jolla, CA) was used to calculate the IC_50_ values.

#### 2.6.2. Cell cycle analysis

Flow cytometry was used to examine the effects of different drug samples on the cell cycle. PC3 cells were treated with the IC_50_ values of the above-mentioned phytosomes. This step was followed by centrifugation to separate the cells after which they were fixed with cold ethanol (70%). The cells were then again separated by centrifugation and washed with phosphate-buffered saline (PBS). Before carrying out the flow cytometric analysis, propidium iodide (PI) was used to stain the cells.

#### 2.6.3. Annexin V staining analysis

An annexin V method was applied to analyze CUR-raw, CUR–PL–SV, and plain PL-SV. Six-well plates were used to grow PC3 cells before incubating them overnight with a variety of treatments at a temperature of 37 °C for 4 h after which the cells were centrifuged. At room temperature, these cells were washed twice and re-suspended in PBS. Thereafter, Annexin V (10 µL) and PI solution (5 µL) was included with the treatments, and cells were incubated at room temperature for 5 min.

#### 2.6.4. B-cell lymphoma 2 (Bcl-2), Bcl-associated X-protein (Bax), p53, caspase 3, nuclear factor kappa beta (NF-kB), and tumor necrosis factor alpha (TNF-α) estimation

Reverse-transcriptase polymerase chain reaction (RT-PCR) was used to express the previously described oncoproteins. IC_50_ concentrations of PL-SV, CUR–PL–SV, and CUR were used to treat the P3 cells. This step was followed by RNA extraction from which the RNA was used to synthesize cDNA. The software named Gene Runner was used to design the primers for the above-mentioned oncoproteins and then β-actin was used for normalizing these samples.

#### 2.6.5. Determination of mitochondrial membrane potential (MMP)

PC3 cells (5 × 10^3^ cells, 96-well plate) were incubated separately with the various treatments for a period of 24 h. The PC3 cells were kept in dark conditions, and tetramethylrhodamine, a methyl ester solution (probe solution), was replaced. A fluorescence-activated cell sorting (FACS) Caliber, BD Biosciences flow cytometer (Franklin Lakes, NJ) was used to detect MMP using the ABCAM assay kit (Cambridge, UK).

### 2.7. Western blot investigation of Bax, p 53, Bcl-2, caspase 3, NF-kB, and TNF_α_ proteins expression

The investigation of optimized formula CUR–PL–SV, CUR-raw, and plain PL-SV formula by Western blot assay was carried according to our laboratory and the previously reported protocols (Mahmood & Yang, 2012; Eid et al., 2022). The band intensity of the Bax, p 53, Bcl-2, caspase 3, NF-kB, and TNF_α_ proteins was normalized vs. the β-actin band intensity (ChemiDoc™ MP imager, Bio-Rad Inc., Hercules, CA).

### 2.8. Statistical analysis

The standard deviation (SD) was used for expression of values. Statistical analysis was performed using a one-way ANOVA and multiple comparisons (Tukey’s) test for which a *p* value of <.05 was deemed significant.

## 3. Results

### 3.1. Design (experimental)

In this study, vesicle size data were fitted into the two-factor interaction (2FI) model, while that of the zeta potential was fitted into the linear model. Notably, for all responses or dependent factors, the fitting model’s forecasted *R*^2^ was reasonably consistent with the adjusted *R*^2^ as shown in Table 3. The adequate precision for both responses was greater than the permissible limit of 4, confirming that the chosen models are pertinent for designing space exploration (Aldawsari & Badr-Eldin, 2020; Alhakamy et al., 2020b).

**Table 3.** Model summary statistics of CUR–PL–SV phytosomes responses.

Responses	Model	Sequential *p* value	*R* ^2^	Adjusted *R*^2^	Predicted *R*^2^	Adequate precision	PRESS	Significant terms
*Y*_1_: vesicle size (nm)	2FI	0.0051	0.9992	0.9987	0.9961	122.4255	112.61	*X*_1_, *X*_2_, *X*_1_*X*_2_
*Y*_2_: zeta potential (mV)	Linear	0.0002	0.9425	0.9234	0.8739	18.4085	70.70	*X*_1_, *X*_2_

CUR: curcumin; PL: Phospholipon^®^ 90H; SV: scorpion venom peptide; PRESS: predicted residual error sum of squares.

**Figure 1.**
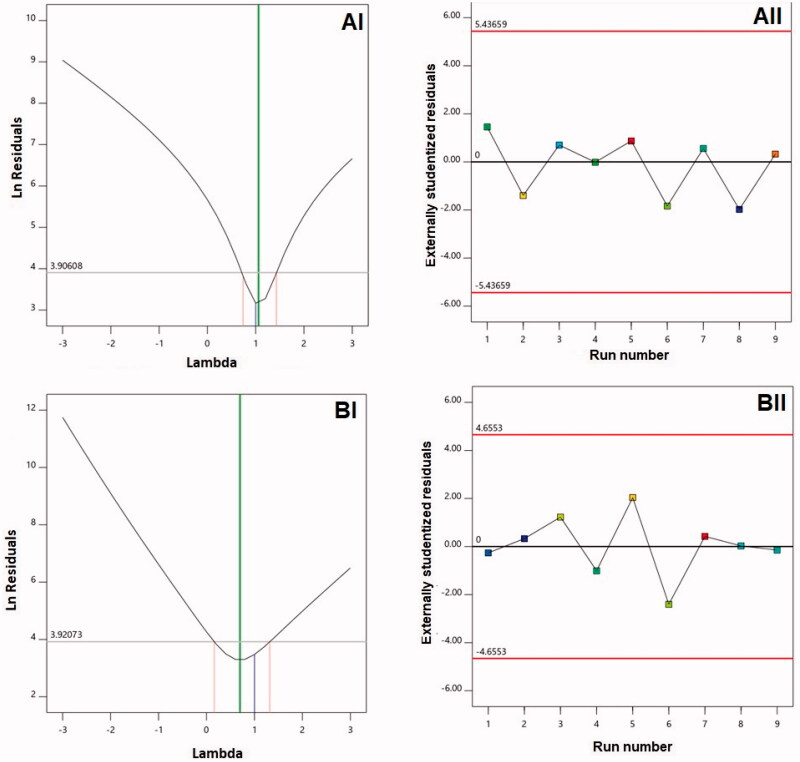
Diagnostic plots for (A) vesicle size and (B) zeta potential of Curcumin-Phospholipon^®^-Scorpion venom (CUR–PL–SV) nano-phytosomes. (I) Box–Cox plot; (II) externally studentized residuals vs. run number plot. CUR: curcumin; PL: Phospholipon^®^ 90H; SV: scorpion venom peptide.

For the two responses, this study developed diagnostic plots to establish the sequential model’s goodness of fit as shown in Figure 1. 1-BI and 1-AI were found to correspond to lambda (*λ*) values of 0.7 and 1.06, respectively. The red lines highlight the confidence limits of 95% comprising the current *λ* value of 1, which indicates no need for particular transformation concerning both responses was present (Badr-Eldin et al., 2021). The zeta potentials and vesicle size ratios of maximum and minimum responses at 9.27 and 2.36, respectively, provide supportive evidence for the absence of transformation. Notably, transformation would be required for a ratio exceeding 10. Figure 1(AII,BII) suggest the absence of any variable that could exert an impact on the responses that are measured (Fahmy et al., 2020).

#### 3.1.1. Effect of variables on vesicle size (Y_1_)

Recently, the focus has shifted to exploring the potential to treat cancer using lipidic vesicle systems. Reportedly, particles <400 nm could be suitable for cancer therapy purposes (Sharma et al., 2014). Accordingly, acceptable vesicle size ranges have been shown by CUR–PL–SV to be 137.5 ± 7.9 to 298.4 ± 11.9 nm. However, nanovesicular system buildup within the tumor and the their effectiveness could be challenged by hindered permeation in pathological situations established by malignancy (Zhang et al., 2019). Thus, reducing vesicle size could enhance tumor penetration (Badr-Eldin et al., 2021). It is for this reason that the nanophytosomal formulation of CUR–PL–SV was optimized to the minimum value. It was suggested that 2FI was the sequential model that related the size of vesicle to the independent variables under investigated. In turn, this process denoted the importance of the primary effects and the contours of inter-variable interactions. The significance of the model could be demonstrated by the model *F*-value of 2005.78. The likelihood that noise would cause the value to be so high was merely 0.01%. The equation shown below was used to denote the model’s 2FI with respect to the factors that were coded:

(1) $$Y_{1}=233.43+64.30\;X_{1}+24.57\;X_{2}-5.18\;X_{1}X_{2}$$

Amounts of SV and PL were found to have a major impact on the size of vesicle (*p*<.0001), according to ANOVA test. Moreover, Table 4 shows that the *X*_1_*X*_2_ interaction was found to show statistical significance (*p*=.0051).

**Table 4.** Analysis of variance (ANOVA) for the vesicle size of CUR–PL–SV nano-phytosomes.

Source	Sum of squares	Degrees of freedom	Mean square	*F*-value	*p* Value
Model	28535.19	3	9511.73	2005.78	<.0001
*X*_1_: PL amount	24806.94	1	24806.94	5231.14	<.0001
*X*_2_: SV amount	3621.13	1	3621.13	763.60	<.0001
*X*_1_ *X*_2_	107.12	1	107.12	22.59	.0051
Residual	23.71	5	4.74		
Cor total	28558.90	8			

**Figure 2.**
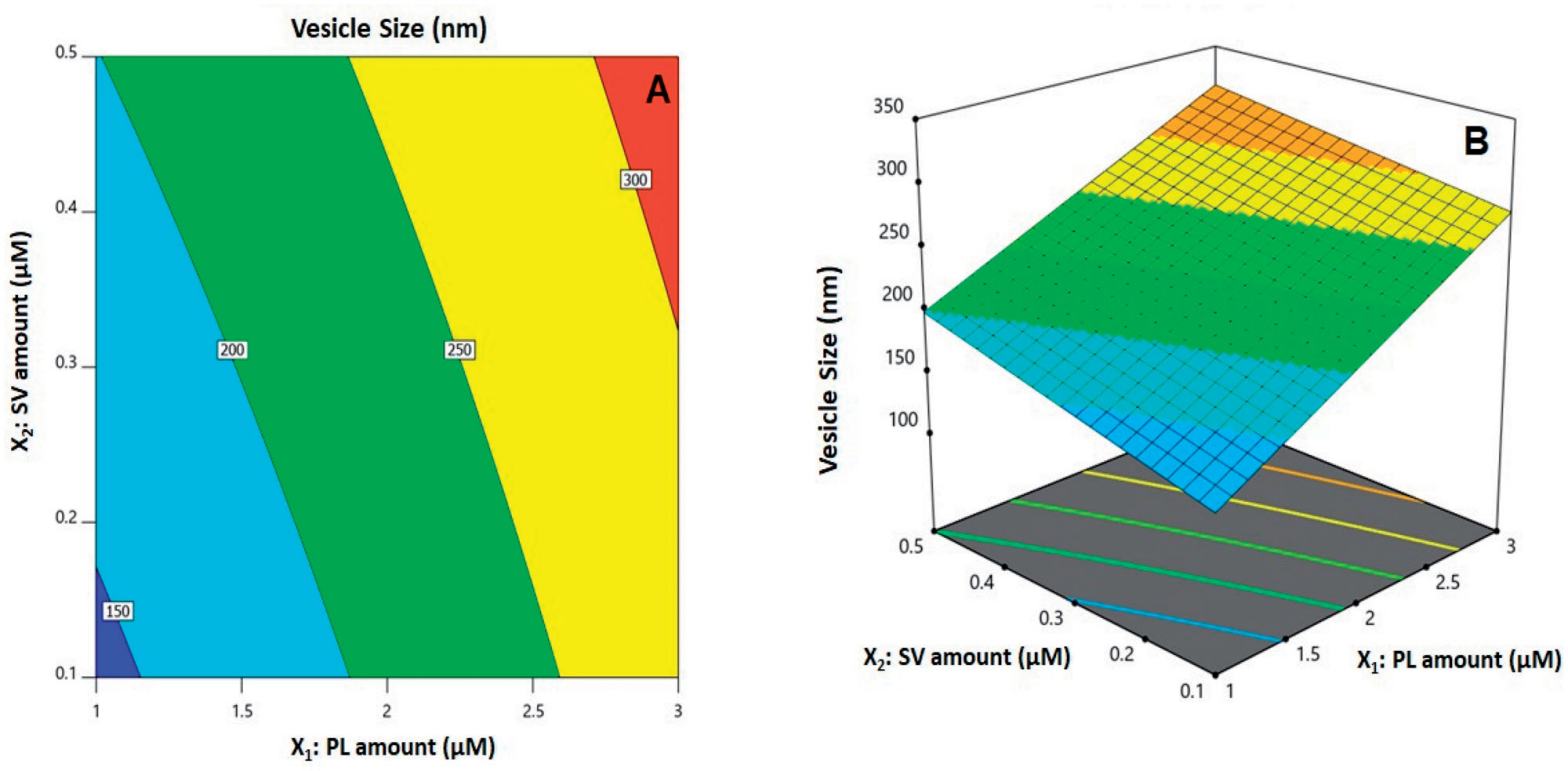
(A) Contour two-dimensional (2D)-plot and (B) response 3D-plot showing the effects and interaction between PL and SV amounts on the vesicle size of CUR–PL–SV nano-phytosomes. CUR: curcumin; PL: Phospholipon^®^ 90H; SV: scorpion venom peptide.

The three-dimensional (3D)-surface plots and 2D-contour plot that show how amounts of SV/PL affected vesicle sizes are illustrated in Figure 2. Evidently, the increased vesicle sizes were attributed to an increase in both *X*_1_ and *X*_2_. In general, the increased size with a corresponding rise in PL quantity agrees with patterns that have been previously reported concerning vesicle system sizes (Saoji et al., 2016; Ahmed & Badr-Eldin, 2019; Zhang et al., 2019). As a case in point, the vesicular sizes of icariin phytosomes with an increased CUR/PL molar ratio have been reported in previous studies (Alhakamy et al., 2020b). At the same time, the SV peptide positive charges could be responsible for the increase in size at greater amounts of SV that is accompanied by heightened vesicular bilayer repulsion caused by the inducement of positive charge. According to prior studies, due to such interventions, the vesicular system (cationic) sizes were reported to be larger than their neutral counterparts (Elsana et al., 2019).

#### 3.1.2. Effect of variables on zeta potential (Y_2_)

The quantitative estimation of nanoparticle system surface systems is denoted by the zeta potential. Cationic nano-vesicles reportedly exhibit a consistent decline in cancerous vessels/tissues in comparison to the adjacent areas (Dubey et al., 2007; Mehanna et al., 2017). In this regard, utilization of SV to induce positive charges in the phytosome surfaces was found to be useful. The surface of all phytosomes (CUR–PL–SV) was found to contain positive charges.

As per the model fit statistics, the zeta potential data fitting the linear model indicating the significance of the main effects of the studied variables is shown in Table 3. The model *F*-value of 49.20 implies the model was significant. Only a 0.02% chance exists that this value could be this high owing to noise. The equation representing the linear sequential model for the zeta potential in terms of coded factors was computed as shown below:

(2) $$Y_{2}=13.80-3.68\;X_{1}+8.63\;X_{2}$$

Statistical analysis with an ANOVA using a sum of squares type III-partial showed that both the amounts of PL (*X*_1_) and SV (*X*_2_) had a significant effect on zeta potentials (Table 5). The effect of the SV quantity was more pronounced than that of the PL quantity as demonstrated by the higher coefficient of the linear term (*X*_2_) and the lowest *p* value.

**Table 5.** Analysis of variance for the zeta potential of CUR–PL–SV phytosomes.

Source	Sum of squares	Degrees of freedom	Mean square	*F*-value	*p* Value
Model	528.61	2	264.30	49.20	.0002
*X*_1_: PL amount	81.40	1	81.40	15.15	.0081
*X*_2_: SV amount	447.21	1	447.21	83.25	<.0001
Residual	32.23	6	5.37		
Cor total	560.84	8			

**Figure 3.**
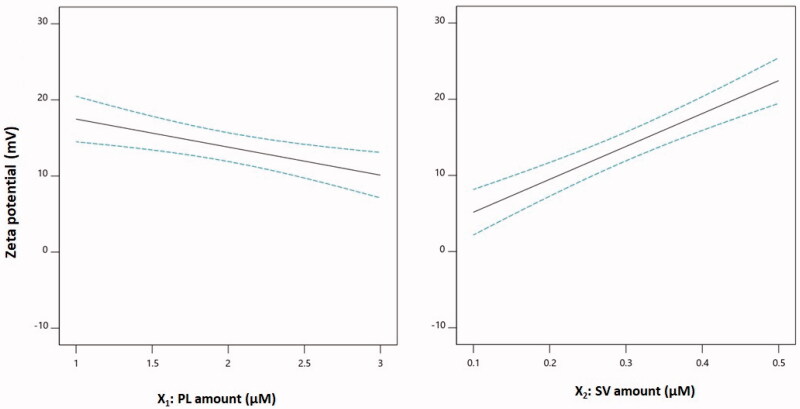
Main effect plots of PL and SV amounts on the zeta potential of CUR–PL–SV phytosomes. CUR: curcumin; PL: Phospholipon^®^ 90H; SV: scorpion venom peptide.

The main effects of both PL and SV amounts on zeta potential are illustrated in Figure 3. The zeta potential decreased with increasing PL amounts, and in contrast, it increased at higher SV quantities. This observation could be supported by the corresponding negative sign of the *X*_1_ term and the positive sign of the *X*_2_ term in the coded equation. The pronounced effect of the amount of SV on the zeta potential could be explained on the basis of the cationic charge of the SV peptides and consequently their role in conferring a positive charge on the phytosomal surface (Wang et al., 2016; Saadat et al., 2019).

#### 3.1.3. Optimization of CUR–PL–SV formulation

The optimized PL and SV levels, with which the set goals of minimizing vesicle size and maximizing zeta potential could be achieved, were predicted by a numerical optimization technique. The optimized amounts of 1.00 and 0.5 µM for PL and SV, respectively, should achieve the objectives when combined with the desirability of 0.800. The measured values for vesicle size and zeta potential of 195.7 nm and 27.3 mV were in good harmony with the predicted values of 198.9 nm and 26.1 mV (Table 6). The relatively low percentage error of less than 5% for both responses confirmed the suitability of the design and the validity of the optimization technique.

**Table 6.** Optimization of CUR–PL–SV phytosomes.

Independent variables	Low	High	Optimum
*X*_1_: PL concentration (µM)	1.0	3.0	1.0
*X*_2_: SV concentration (µM)	0.1	0.5	0.5

Responses	Predicted value	Observed value
*Y*_1_: particle size (nm)	198.9	195.7
*Y*_2_: zeta potential (mV)	26.1	27.3

CUR: curcumin; PL: Phospholipon^®^ 90H; SV: scorpion venom peptide.

### 3.2. Evaluation of anticancer potential of CUR–PL–SV phytosomes

#### 3.2.1. Determination of IC_50_ values

**Figure 4.**
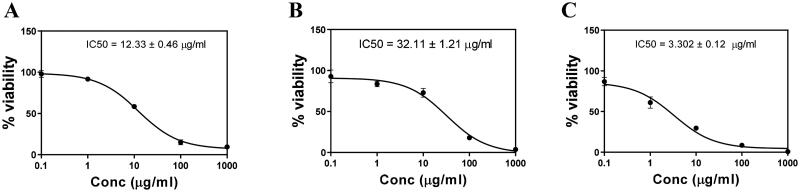
Cytotoxicity of (A) PL-SV, (B) CUR, and (C) CUR–PL–SV estimated by the MTT assay. Based on these results, the IC_50_ of samples was calculated after the 48 h treatment using an MTT assay. Data were calculated as the mean of four independent experiments ± SD. CUR: curcumin; PL: Phospholipon^®^ 90H; SV: scorpion venom peptide.

The cytotoxic potential positively correlated to the anticancer potential of drug. When PC3 cell line was exposed to the PL-SV, CUR, and CUR–PL–SV as shown in Figure 4, CUR–PL–SV showed potent cytotoxicity at the lowest concentration compared to PL-SV and CUR. The results showed that the IC_50_ values were 12.33 ± 0.46, 32.11 ± 1.21, and 3.302 ± 0.12 µg/mL for PL-SV, CUR, and CUR–PL–SV, respectively. It should be noticed that the IC_50_ value of CUR–PL–SV refers to equivalent amount of CUR in the nanovesicle formula and the IC50 value of PL-SV refers to equivalent amount of SV in Pl-SV.

#### 3.2.2. Cell cycle analysis

**Figure 5.**
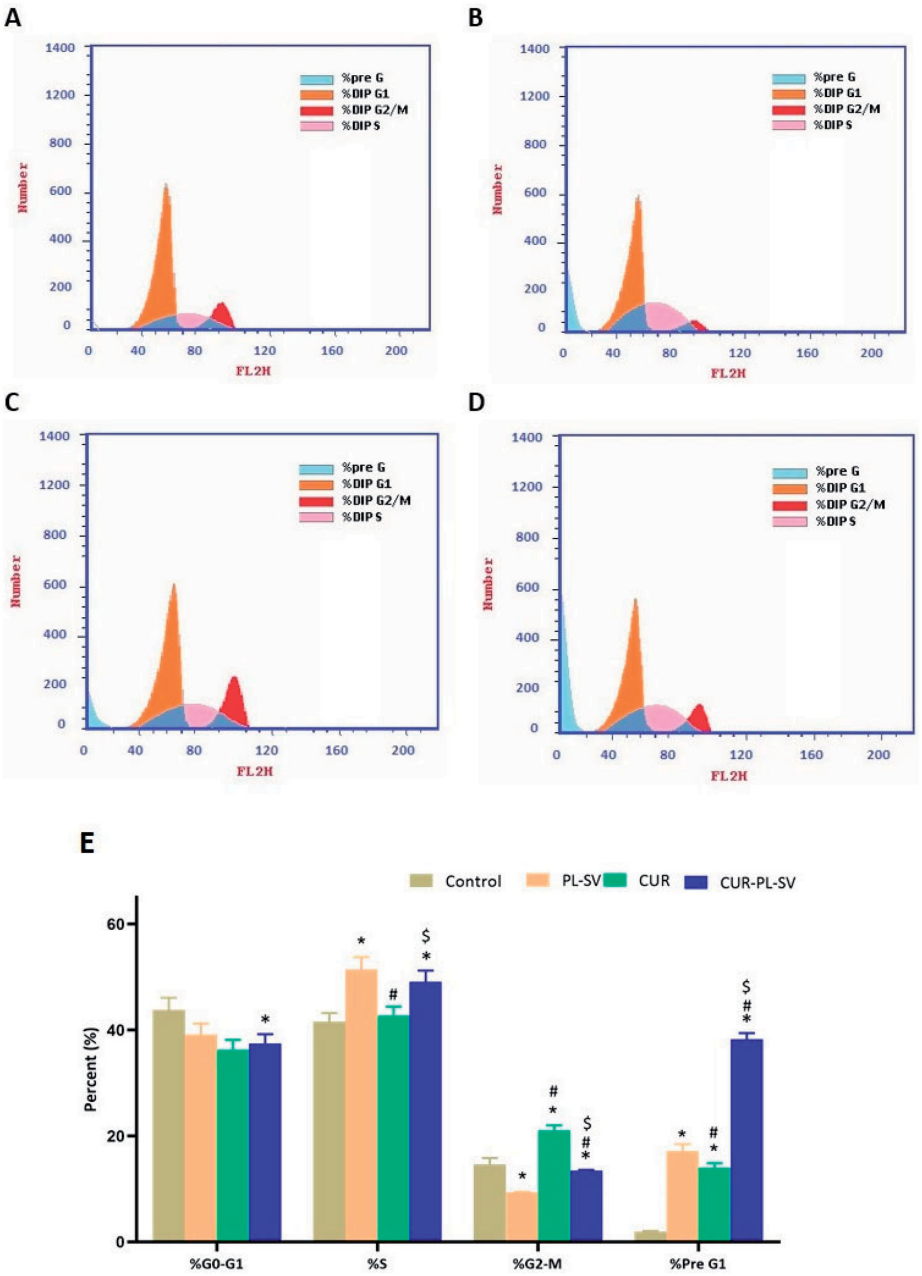
Cell cycle analysis by flow cytometry. (A) Untreated Control, (B) PL-SV, (C) CUR, (D) CUR–PL–SV, and (E) bar diagram of the different cycle phases. Data are the mean of four independent experiments ± SD. *Significantly different vs. control, *p*<.05; ^#^significantly different vs. PL-VS, *p*<.05; ^$^significantly different vs. CUR. CUR: curcumin; PL: Phospholipon^®^ 90H; SV: scorpion venom peptide.

The outcome of cell cycle analysis showed that cells of the control group were present at a maximum in the G0–G1 and S phases with optimum proliferation. The percentages of cells present in the G2-M and pre-G1 phases signify apoptotic activity or cell cycle arrest. Thus, it can be observed from Figure 5 that CUR–PL–SV produced potent anticancer effects against PC3 cell line via cell cycle arrest in the pre-G1 phase compared to the effects produced by CUR and PL-SV individually. However, CUR showed a superior anticancer impact on the G2-M phase compared to CUR–PL–SV. However, overall, CUR–PL–SV showed superior anticancer potential and arrested cell cycle and hence, inhibited proliferation. The percentage of data was estimated after excluding dead cells in the pre-G phase.

#### 3.2.3. Annexin V assay for apoptotic activity

**Figure 6.**
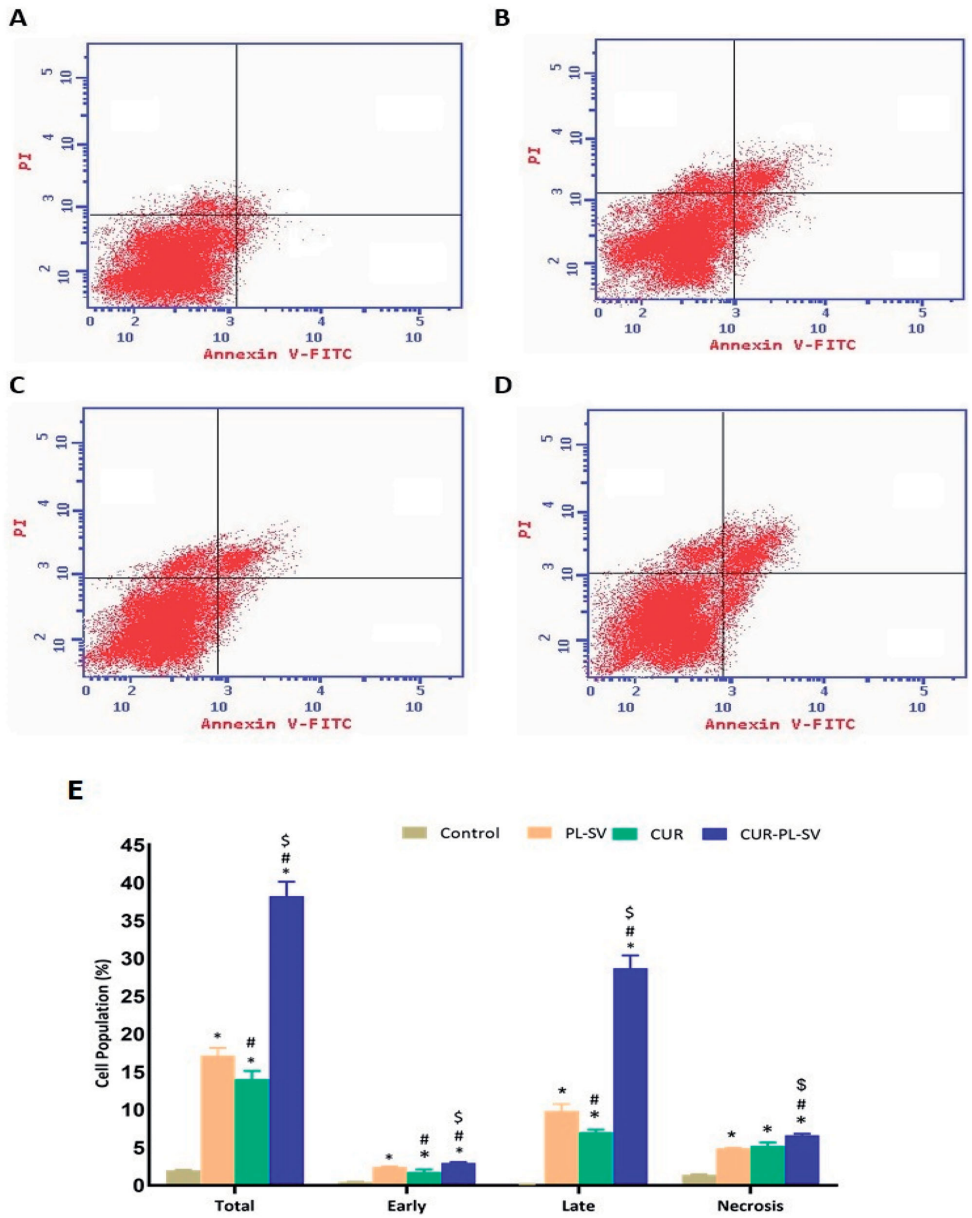
Annexin assay for apoptotic activity. (A) Untreated control, (B) PL-SV, (C) CUR, (D) CUR–PL–SV, and (E) bar diagram of different types of cell death. Data are the mean of four independent experiments ± SD. *Significantly different vs. control, *p*<.05; ^#^significantly different vs. PL-VS, *p*<.05; ^$^significantly different vs. CUR. CUR: curcumin; PL: Phospholipon^®^ 90H; SV: scorpion venom peptide.

Based on the annexin V assay, it was found that treatment of PC3 cells with CUR–PL–SV caused an increase in early, late, and total percentages of apoptotic cells as represented in Figure 6. The findings also validated the success of the CUR-SV phytosome as this phytosome showed superior apoptotic activity compared to CUR and PL-SV. Additionally, the percentage of apoptotic cells in the late phase was significantly higher than in the other phases. Moreover, CUR–PL–SV also showed increased necrosis, whereas PL-SV and CUR showed similar effects against the necrosis of PC3 cells.

#### 3.2.4. Expression of Bax, caspase-3, p53, and Bcl2

**Figure 7.**
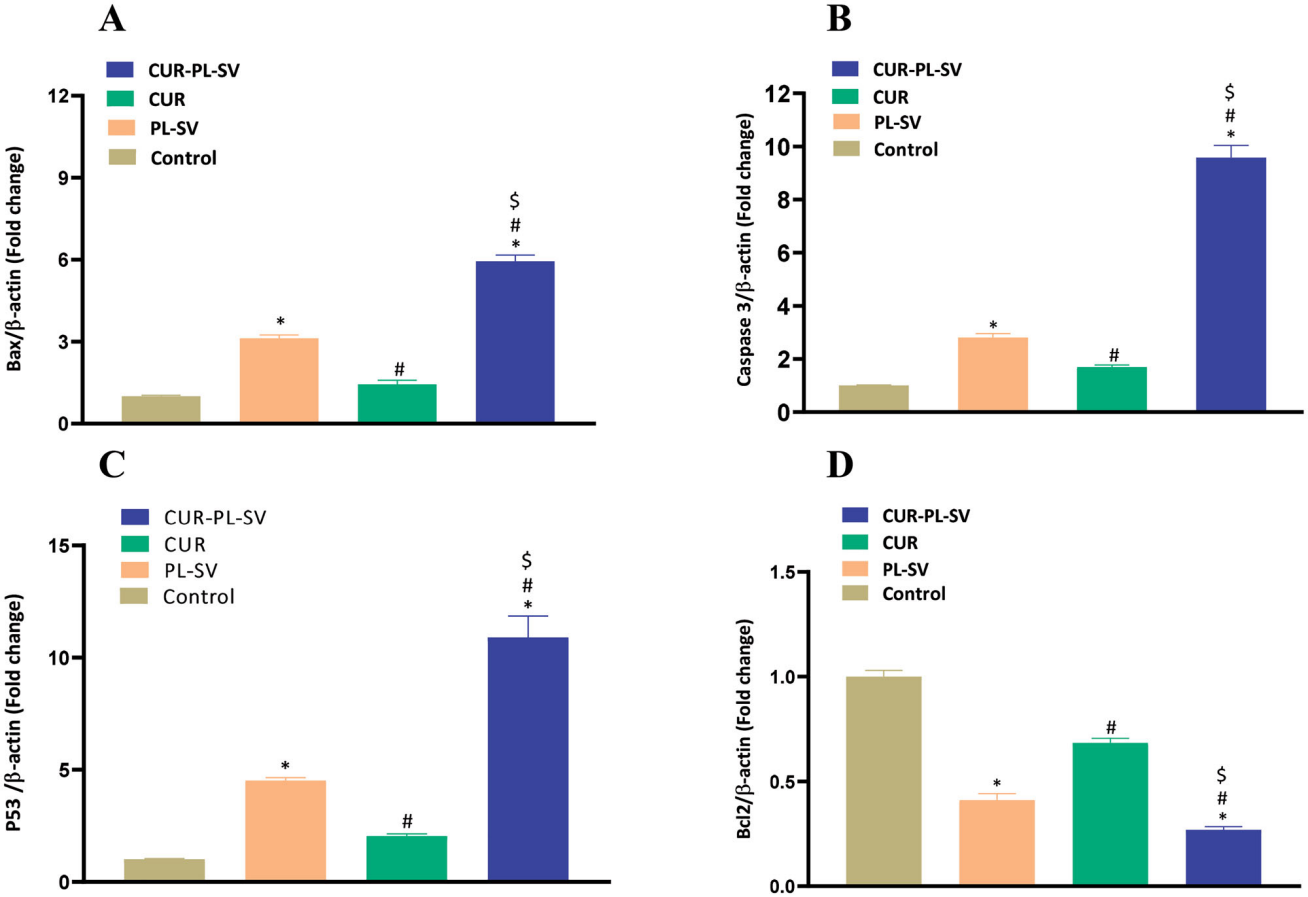
Expression of apoptotic markers (A) Bax, (B) caspase-3, (C) p53, and (D) Bcl-2. CUR: curcumin; PL: Phospholipon^®^ 90H; SV: scorpion venom peptide. Data are the mean of four independent experiments ± SD. *Significantly different vs. control, *p*<.05; ^#^significantly different vs. PL-VS, *p*<.05; ^$^significantly different vs. CUR. CUR: curcumin; PL: Phospholipon^®^ 90H; SV: scorpion venom peptide.

Apoptosis is one of the most important parameters for validating the anticancer potential of a drug. The expression level of pro- and anti-apoptotic proteins signifies the rate of apoptosis. In this study, treatment of PC3 cells with CUR–PL–SV led to an increase in the expression level of Bax, caspase-3, and p53 and hence induced apoptosis. Additionally, CUR–PL–SV also led to a reduction in the expression of the anti-apoptotic protein, Bcl-2 and thus, confirmed the occurrences of apoptotic and anticancer activities compared to PL-SV and CUR as shown in Figure 7. Control incubations did not receive any treatment.

#### 3.2.5. Expression of TNF-α and NF-kB

**Figure 8.**
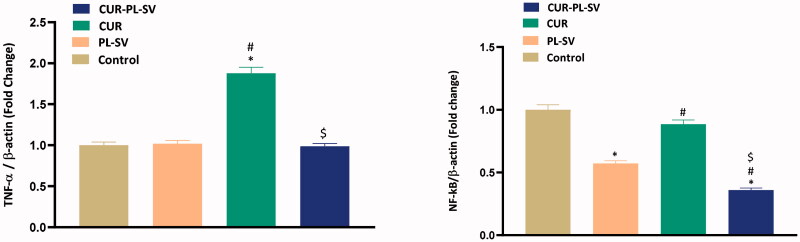
Expression level of inflammatory markers (A) TNF-α and (B) NF-kB. Data are the mean of 4 independent experiments ± SD. *Significantly different vs. control, *p*<.05; ^#^significantly different vs. PL-VS, *p*<.05; ^$^significantly different vs. CUR. CUR: curcumin; PL: Phospholipon^®^ 90H; SV: scorpion venom peptide.

NF-kB is a well-known transcription factor and, once activated, it regulates the transcription of various proinflammatory cytokines, such as TNF-α. Increased expression of NF-kB and TNF-α promotes tumor initiation, progression, and angiogenesis. In the present study, Figure 8 shows that when PC3 cells were treated with CUR–PL–SV, significant reductions in the expression of activated NF-kB and TNF-α were found thus signifying the anticancer potential of CUR–PL–SV. Control incubations did not receive any treatment.

#### 3.2.6. Mitochondrial membrane potential study

**Figure 9.**
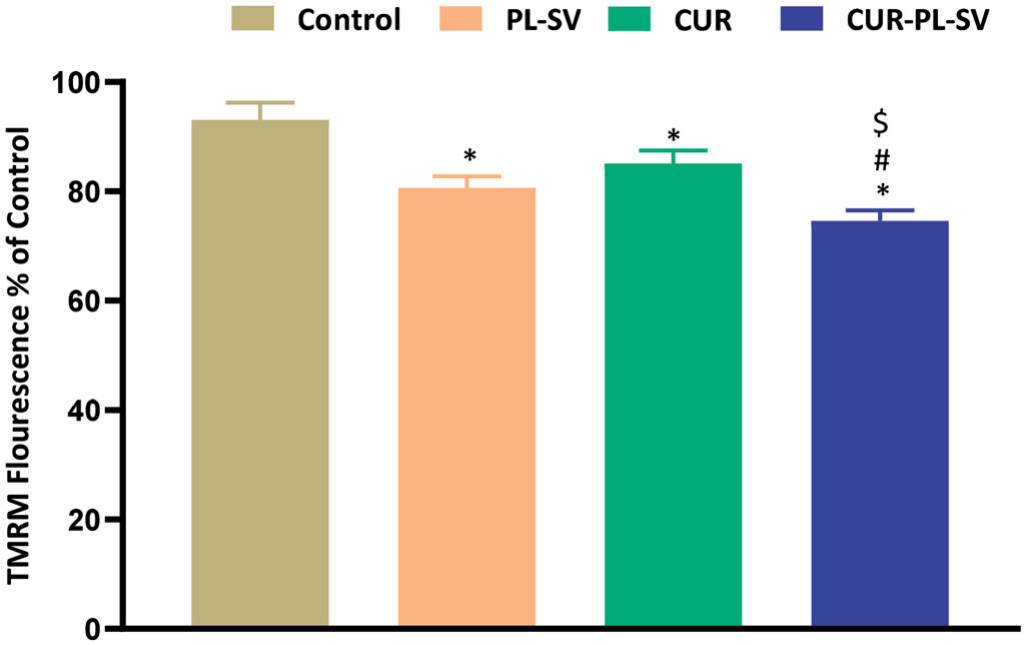
Change in mitochondrial membrane permeability. Data are the mean of four independent experiments ± SD. *Significantly different vs. control, *p*<.05; ^#^significantly different vs. PL-VS, *p*<.05; ^$^significantly different vs. CUR. CUR: curcumin; PL: Phospholipon^®^ 90H; SV: scorpion venom peptide.

Acquisition of tetramethylrhodamine-6-maleimide (TMRM) into mitochondria is a predictive pointer of MMP and provides substantial information about apoptotic activity. High retention of fluorescence signifies healthy mitochondria and mitochondrial membranes and vice versa. In the present study, when PC3 cells were treated with CUR–PL–SV and studied for the MMP, it was found that CUR–PL–SV caused a significant reduction in the percentage of fluorescence compared to other treatment groups and signified a reduction in mitochondrial membrane potential and also provided substantial evidence for the superior apoptotic activity of CUR–PL–SV compared to PL-SV and CUR as shown in Figure 9.

### 3.3. Western blot assay

**Figure 10.**
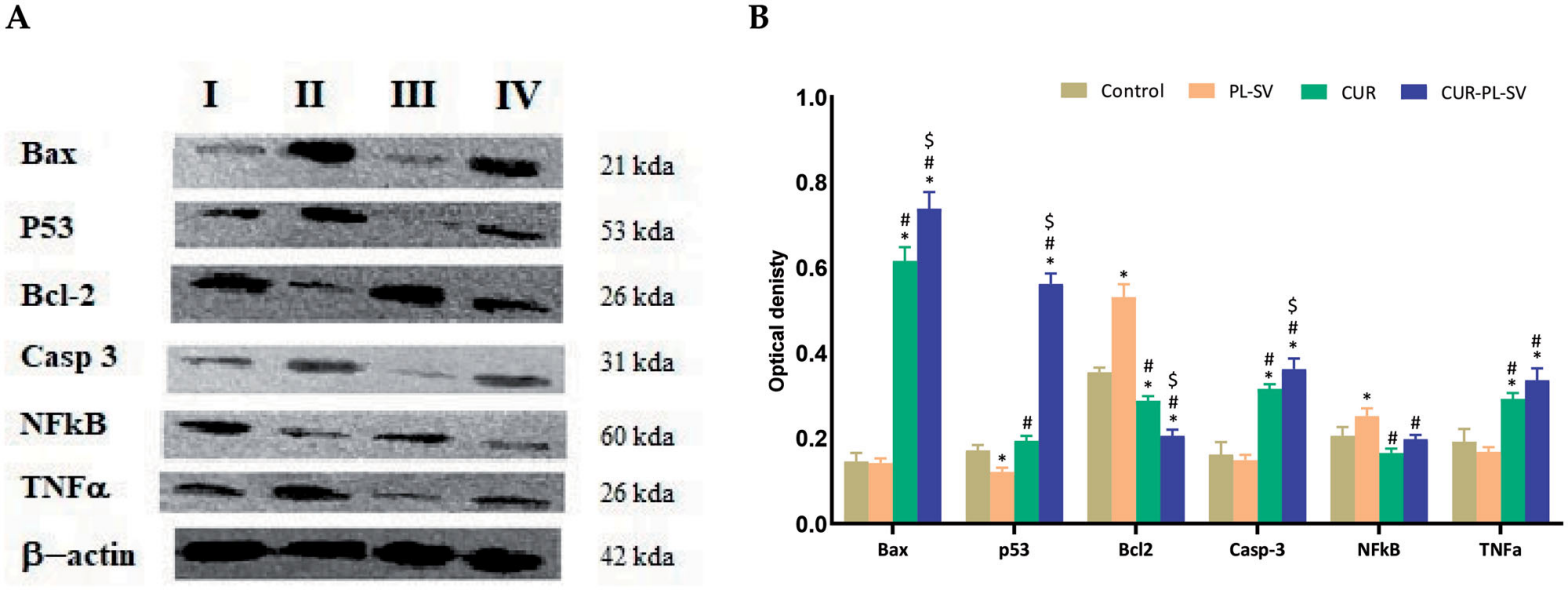
Western blots (A) and histogram for proteins expression (B) of Bax, p 53, Bcl-2, caspase 3, NF-kB, and TNFα protein expression for the four groups in PC3 cells; (I) control, (II) CUR–PL–SV, (III) PL-SV, and (IV) CUR. Data are the mean of three independent experiments ± SD. *Significantly different vs. control, *p*<.05; ^#^significantly different vs. PL-VS, *p*<.05; ^$^significantly different vs. CUR. CUR: curcumin; PL: Phospholipon^®^ 90H; SV: scorpion venom peptide.

Western blot assay was carried out for the investigation of the effect of the optimized formula CUR–PL–SV, CUR-raw, and plain PL-SV formula on Bax, p 53, Bcl-2, caspase 3, NF-kB, and TNFα protein expression. The results revealed that the optimized CUR–PL–SV formula showed a significant (*p*<.05) increase in Bax, P53, and caspase 3 protein expression when compared with control, CUR-raw, and plain PL-SV formulas (Figure 10(A,B)).

## 4. Discussion

Recently, the occurrence and prevalence of PC, has increased considerably (Sung et al., 2021). Ideally, PC is diagnosed at the old stage, and the use of the various chemotherapeutic drug is associated with severe side effects. Moreover, diagnosis is usually done at a later stage, and hence, the survival rate is compromised (Litwin & Tan, 2017). Recently, various targeted therapies and immunotherapy modalities have been used, but the clinical outcomes and their cost are not up to the mark (Litwin & Tan, 2017). Thus, various bioactive natural sources have been explored for the possible anticancer potential (Wang et al., 2008; Liu et al., 2018). Among various natural products, CUR has been extensively explored for anticancer potential in preclinical and clinical setup (Hong et al., 2006; Teiten et al., 2010; Baldi et al., 2020; Liczbiński et al., 2020). CUR is categorized as ‘GRAS’ by the US FDA, and hence its safety is well assured (Carlson, 2021). Despite being a potent natural bioactive, CUR is poorly soluble, possesses low bioavailability, and undergoes rapid hepatic metabolism. SV is another protein-based natural product and is reported to possess potent anticancer potential (Ortiz et al., 2015; Al-Asmari et al., 2016, 2018; Akef et al., 2017; Rapôso, 2017; Gómez Rave et al., 2019). However, unlike CUR, SV also suffers from the limitation of low thermal stability, short half-life and poor controlled release pattern (Ortiz et al., 2015).

Therefore, in the present study, the potential of anticancer activity of CUR and SV was investigated. In this study, SV-conjugated CUR phytosomes were fabricated and studied for their anticancer potential using the PC3 cell line after which various cellular and molecular parameters were examined. PC3 cells do not express androgen receptor (androgen-independent) or prostate-specific antigen (Kaighn et al., 1979; Van Bokhoven et al., 2003). PC3 cells show highly aggressive behavior representing the aggressive forms of prostatic adenocarcinoma and castration-resistant tumors (Tai et al., 2011). PC3 cells have been used extensively by researchers to represent the aggressive form of PC. PC3 cells have the characteristics of prostatic small cell neuroendocrine carcinoma (Tai et al., 2011).

In this work, identification of the variables that affect CUR–PL–SV nanovesicle characteristics is essential. The factorial design offers an advantage concerning this issue as it possesses the ability to analyze the influence of different factors concurrently. SV peptides are K(+)-channels blockers and/ or Na(+)-channel modifiers (Gao et al., 2013; Ortiz et al., 2015). Phytosomes showed superior advantage of enhancing the pharmacokinetic properties and hence improving the bioavailability of encapsulated natural therapeutic agents when compared with liposomes (Aguirre et al., 2011; Jemal et al., 2011). The fabricated phytosomes were experimentally optimized and characterized in terms of vesicle size and zeta potential. Vesicle size is one important parameter that determines the anticancer potential of phytosome in the tumor microenvironment. A vesicle size <400 nm is considered ideal for anticancer activity. In the present study, vesicle size of the optimized formula was 195.7 nm. Apart from the vesicle size, surface charge is a decisive parameter necessary for penetration of the tumor mass. Cationic nanovesicles are considered ideal for tumor penetration. Thus, the positive charge of prepared CUR–PL–SV potentiated the anticancer property of the prepared phytosome as a result of the positive charges on the SV peptide (Ortiz et al., 2015). The optimized formula showed a zeta potential value of 27.3 mV.

After the successful characterization of fabricated CUR–PL–SV, we validated the anticancer potential of the prepared phytosome. First, an MTT assay was performed to validate the cytotoxic potential. When PC3 cells were treated with CUR–PL–SV, CUR, and PL-SV, CUR–PL–SV showed enhanced cytotoxicity at a much lower concentration than PL-SV and CUR. Uncontrolled cell division is a major hallmark of carcinogenesis (Balk & Knudsen, 2008). Any drug that arrests the cell cycle is considered an ideal anticancer drug (Balk & Knudsen, 2008). The cell cycle data revealed that CUR–PL–SV predominantly induced cell cycle arrest at pre-G1 and G2-M phases and hence confirmed the anticancer potential of phytosomes. Apoptosis is one of the critical parameters for the determination of the anticancer potential of drug candidates (Ali & Kulik, 2021). The initial work for the association between apoptosis and carcinogenesis was established in the 1970s (Wong, 2011). Reduced apoptosis is commonly seen during carcinogenesis (Ali & Kulik, 2021). Apoptosis is regulated by the presence of pro- and antiapoptotic proteins. Bax, BAD, and caspases are considered as pro-apoptotic proteins, and Bcl-2 is a well-studied antiapoptotic protein (Lu et al., 2020). In the case of PC and other cancer, reduced or inhibited apoptosis and evasion of apoptosis were reported. Additionally, tumor cells inhibit the level of various pro-apoptotic proteins and increase the level of antiapoptotic protein (Lu et al., 2020). Thus, any drug candidate that induces apoptosis in tumor cell is considered a potent anticancer agent. In our study, when the PC3 cells were treated with CUR–PL–SV, CUR, or PL-SV, CUR–PL–SV showed enhanced induction of early late and total apoptosis and necrosis. The results also revealed enhanced cytotoxic effects of SV on PC3 cells when compared with CUR. SV has been previously reported to induce apoptosis in less sensitive tumor cells with no effect on normal cell lines, such as MRC-5, MDCK, and Vero (Díaz-García et al., 2013).

Additionally, an RT-PCR assay also confirmed the superior apoptotic activity of CUR–PL–SV where increased expression of Bax and caspase-3 and reduced expression of Bcl-2 were found. To confirm the mechanism of apoptosis, we also checked the expression level of p53. p53 is an extensively studied tumor suppressor gene, and in the case of PC, its expression level was significantly increased (Chi et al., 1994). p53 also acts as an important checkpoint during the uncontrolled cycle and causes cell cycle arrest via modulation of Rb, CDK, and other cell cycle machinery components (Chi et al., 1994). In the present study, we found a significant elevation in the expression level of p53 when PC3 cells were treated with CUR–PL–SV as compared to CUR and PL-SV.

It is also important to understand that the mitochondria are important organelles that regulate apoptotic activity. In general, mitochondria are sites for oxidative phosphorylation and ATP production, but these organelles are also involved in the apoptotic process (Armstrong, 2006). In response to the increased level of Bax, holes are produced on the mitochondrial surface in response to reduced MMP, and cytochrome C is released, which in combination with Apaf and AIF, form apoptosomes and ultimately produces caspase 3 (Tsujimoto, 1998; Armstrong, 2006; Wang & Youle, 2009). Thus, a potent anticancer drug should reduce the MMP so that the apoptotic cascade can begin. Interestingly, in our study, a significant reduction in the MMP when PC3 cells were treated with CUR–PL–SV compared to CUR and PL-SV was found.

TNF-α promotes apoptosis through NF-kB (Chipuk et al., 2004). TNF-α binds with the type I cell surface receptor (TNFRI) that can activate the NF-κB survival pathway or caspase-dependent cell death (Harper et al., 2003; Micheau & Tschopp, 2003). NF-kB is a major transcription factor that regulates the production of proinflammatory cytokines (Schmitt & Zischka, 2018; Iqubal et al., 2020a; Piotrowski et al., 2020). NF-kB is ideally located in the cytoplasm, and in response to tumor stimulus, it undergoes nuclear translocation and regulates the transcription of various proinflammatory cytokines and interleukin (Iqubal et al., 2018, 2020b,c; Tuli et al., 2021). Studies have shown that increased activation and nuclear translocation are associated with tumor initiation, invasion, angiogenesis, and metastasis (Gupta et al., 2010; Gómez-Valenzuela et al., 2021). Thus, in the present study, the expression levels of NF-kB and TNF-α were also assessed. Our finding showed reduced expression of activated NF-kB and TNF-α and thus confirmed the anti-inflammatory in addition to the anticancer potential of CUR–PL–SV.

## 5. Conclusions

In the present study, SV conjugated CUR phytosomes were fabricated, optimized, and characterized. The fabricated phytosomes showed reduced vesicle size and positive surface charge, which is critical for optimum anticancer activity. Fabricated phytosome was studied for cell cycle arrest, apoptosis stimulation, mitochondrial membrane permeability, and inflammatory mediator. Cellular and molecular analysis using human prostatic cancer PC3 cells showed arrest of the cell cycle at the pre-G1 phase, enhanced apoptosis and necrosis, reduced mitochondrial membrane permeability, reduced Bcl-2, NF-kB, TNF-α levels, and increased Bax, caspase, and p53 levels upon treatment with SV conjugated CUR phytosomes.
